# Young honeybees show learned preferences after experiencing adulterated pollen

**DOI:** 10.1038/s41598-021-02700-6

**Published:** 2021-12-02

**Authors:** Rocío Lajad, Emilia Moreno, Andrés Arenas

**Affiliations:** 1 Laboratorio de Insectos Sociales, Departamento de Biodiversidad y Biología Experimental, Facultad de Ciencias Exactas y Naturales, Universidad de Buenos Aires, Buenos Aires, Argentina; 2 Instituto de Fisiología, Biología Molecular y Neurociencias (IFIBYNE), CONICET – Universidad de Buenos Aires, Buenos Aires, Argentina

**Keywords:** Cognitive neuroscience, Learning and memory, Olfactory system, Social behaviour, Animal behaviour, Animal physiology, Entomology, Neuroscience, Zoology

## Abstract

Pollen selection affects honeybee colony development and productivity. Considering that pollen is consumed by young in-hive bees, and not by foragers, we hypothesized that young bees learn pollen cues and adjust their preferences to the most suitable pollens. To assess whether young bees show preferences based on learning for highly or poorly suitable pollens, we measured consumption preferences for two pure monofloral pollens after the bees had experienced one of them adulterated with a deterrent (amygdalin or quinine) or a phagostimulant (linoleic acid). Preferences were obtained from nurse-aged bees confined in cages and from nurse bees in open colonies. Furthermore, we tested the bees’ orientation in a Y-maze using a neutral odour (Linalool or Nonanal) that had been previously associated with an amygdalin-adulterated pollen. Consumption preferences of bees, both in cages and in colonies, were reduced for pollens that had been adulterated with deterrents and increased for pollens that had been supplemented with linoleic acid. In the Y-maze, individuals consistently avoided the odours that they had previously experienced paired with the deterrent-adulterated pollen. Results show that nurse-aged bees associate pollen-based or pollen-related cues with either a distasteful/malaise experience or a tasty/nutritious event, leading to memories that bias their pollen-mediated response.

## Introduction

The success of animals depends on their ability to select the most appropriate food from the resources available at a given time and to predict food in a complex environment, processes that rely strongly on learning and memory. This is also true among social insects whose colonies respond flexibly to changing conditions. When searching and collecting resources outside the nest, foraging workers assess the value of the food and subsequently associate odours, colours, textures, and other features of the food source with the reward it provides^1–6^. In social insects, learning about source features occurs not only in the field, but also within the nest. For instance, when resources foraged outside are further processed inside by a different group of workers^7–9^. Such social learning is relevant to generate an adaptive collective response as individuals engage in different tasks, learn, and respond to information about available resources according to their own experiences and requirements^10^. The question of how food information is learned by different groups of workers is particularly interesting in honeybees because pollen is consumed by the nurse bees inside the nest and not by the foragers that collect it.

In the honeybee *Apis mellifera* L., division of labour is associated with the age of the workers, who shift from in-hive tasks as young bees to searching and collecting resources outside the hive as they age. Initially, workers clean the comb cells before they move on to brood care and food handling and storage. After three weeks of adult life, workers initiate foraging^11,12^. Due to differences in the physiological states among nest mates and their distinct nutritional needs, in-hive workers have access to different resources than foragers, which makes them prime candidates for assessing, learning and memorising information about resources like pollen.

Pollen is the main protein and lipidic source for bee colonies. Depending on their botanical origin, pollens can strongly differ in their composition^13–15^ and may present toxic compounds^16–20^, phago-deterrents^17,21^, and/or digestibility-altering compounds^16^. Pollen is collected by specialized foragers that transport it into the nest using external structures located on the third pair of legs (i.e., corbiculae). Foragers do not ingest fresh pollen during collection. Once inside the nest, fresh pollen is largely handled and ingested by in-hive young workers^22,23^ of 3–4 to 8–10 days of age. These workers act as nurse bees, digesting most of the available pollen and using it to feed the larvae, queen, drones, and the rest of the workers with a protein-rich jelly by means of trophallaxis^10,24–26^.

In the field, pollen selection might be based on the foragers’ innate preferences^27–30^ to pollen-based cues (i.e., pollen odour, colour, and taste) and on their previous experience with neutral cues associated to pollen sources (henceforth: pollen-related cues^31–33^). However, it is likely that pollen selection by foragers may be limited to the perception of pre-ingestive pollen cues (foragers do not consume fresh pollen at the source)*.* Consistently, studies show that foragers are not able to make foraging decisions solely based on the presence of nutrients (e.g., proteins or lipids^34^) or toxic compounds^20,35^ that can only be assessed after pollen ingestion^13,36–39^. On the contrary, young bees do exhibit preferences for high-quality pollens^40,41^ and avoid toxic ones^16^. The extent to which young workers are able to learn species-specific pollen cues according to their own assessment and experience with the resource remains unexplored in honeybees.

Considering that nurse bees might be making decisions regarding which pollen to use to nourish the colony, we studied whether their behaviour is plastic enough to mediate the selection of pollen. We hypothesized that young bees can associate species-specific pollen-based and/or neutral pollen-related cues with nutritional or non-nutritional compounds, when pollen is used as a reinforcement^33^. We expected tasty and nutritious substances (e.g., linoleic acid, an essential fatty acid with phage-stimulating properties^42,43^) to reinforce learning positively, and substances that make pollen unpalatable or harmful to bees (e.g., amygdalin or quinine that yield significant post-ingestional mortality in bees^17,44^) to reinforce learning negatively. Thus, appetitive and aversive experiences would turn into memories enabling nurse bees to predict pollen attributes using previously learned informational cues.

We investigated the ability of young honeybees to learn and memorize pollen cues according to changes in resource quality (e.g., increase in nutritional content or toxicity, increase/decrease in palatability). In the first experiment, we tested whether nurse-aged bees confined in cages could learn to discriminate between two pure pollens: one that they had previously experienced adulterated with amygdalin, quinine, or linoleic acid and another one that they had experienced unadulterated. Measuring pollen consumption, we predicted that bees would discriminate between pollens using learned pollen-based cues. They would prefer less those that had been paired with the deterrent substances and would prefer more those that had been supplemented with linoleic acid. In the second experiment, we tested whether nurse-aged bees could associate a neutral cue (namely an olfactory cue) with pollen (adulterated or not) and, in turn, use that cue to predict resource suitability. We predicted that memories established after experiencing a neutral odour (Linalool or Nonanal) paired with amygdalin-adulterated pollen would reduce orientation preferences for the learned odour when tested against a novel odour in a Y-maze one day later. In the third experiment, we assessed whether the learned preferences for pollen-based cues studied in experiment 1 (i.e., using caged nurse-aged bees and only two types of pollen) could also be observed in the social context of the hive and with a wider range of pollens.

## Materials and methods

### Study site and bees

**Figure 1:**
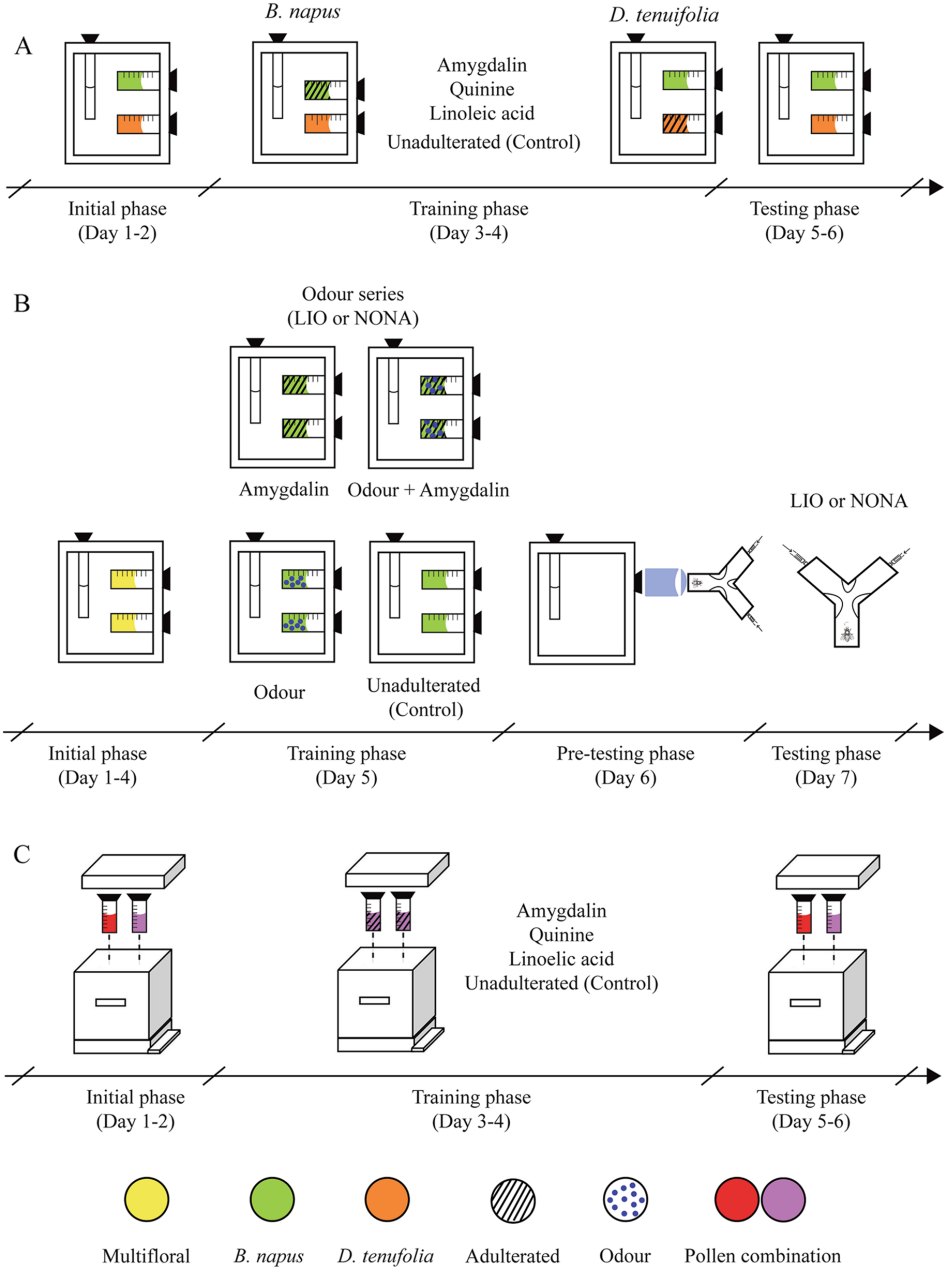
Schematic schedule of the experiments. Procedures followed in experiments 1 (**A**), 2 (**B**) and 3 (**C**) showing: the experimental phases (and the days when they took place), the pollens involved, whether or not pollens were adulterated and/or scented and, in (**C**), the Y-maze used for testing. In (**A)** and (**B)** sugar solution was offered ad libitum through plastic tubes.

We performed the experiments during the summer seasons of 2018/2019 and 2019/2020 in the Experimental Field of the School of Exact and Natural Sciences of the University of Buenos Aires (34°32′S, 58°26′W). Bees for experiments 1 and 2 emerged from brood frames (Fig. S1) that had been placed two days earlier in an incubator at 34 ºC, 55% relative humidity, and darkness. Emerging zero/1-day-old workers were manually collected and placed in wooden cages (10 × 10 × 10 cm) in groups of 80–90 individuals. The experiments started immediately after collection, following the schedule shown in Fig. 1A, B. Bees were fed 50% w/w sugar solution ad libitum during all the experimental period. We used a total of 40 cages for experiment 1 and 48 cages for experiment 2. Replicates of each treatment came from frames from different hives. In experiment 3, we used 24 open colonies of about 15,000 worker bees each, all containing a mated queen, 4–5 brood frames, and 1–2 frames with food reserves.

### Pollen quality modification

To assess the ability to learn and retain pollen information after positive or negative experiences, we compared learned preferences for singular combinations of two pollens, one of which had been previously adulterated and another one that had been kept unadulterated (Control). We adulterated pollens to modify their suitability for the bees. To reduce pollen quality, we added amygdalin (0.1 M) or quinine (0.1 M), both deterrent substances that yield significant post-ingestional mortality in bees^44^. To improve pollen quality, we added 4% w/w of conjugated linoleic acid (CLA), an essential fatty acid with phagostimulant activity^42,43,45,46^. We did not work with nor manipulate plants; the pollen we used was collected naturally from plants by bees. For the experiments, we used monofloral pollens obtained from multifloral or monofloral bee-collected samples (provided and characterized by the Pampero, Amuyen, and CoopSol cooperatives). In the case of multifloral samples, we separated the pollen pellets according to their botanical origin based on their colour, brightness, texture and degree of agglutination. Most pollen samples were obtained during the same season as that of data collection. We crushed, weighed and hydrated pollens to obtain a bee bread-like paste which we offered *al libitum* using individual tipless 5 ml graduated syringes for each monofloral pollen (Fig. S1).

### Experiment 1: Testing learned preferences in nurse-aged bees that experienced changes in the quality of the offered pollens

To test the extent to which nurse-aged bees adjust their preferences to the most suitable pollens, we measured consumption preferences towards *Brassica napus* L. and *Diplotaxis tenuifolia* (L.) DC. pollens of bees that had previously experienced one of them altered with amygdalin, quinine or linoleic acid. A priori, we expected that bees with previous experience would avoid consuming the pollen associated with amygdalin or quinine, and would increase consumption of the pollen that had been previously supplied with linoleic acid.

The experiment lasted for 6 days and consisted of three different phases: *initial*, *training*, and *testing* (Fig. 1A). During the whole experiment, caged bees received both *B. napus* and *D. tenuifolia* pollens simultaneously but provided in individual feeders. During the *initial phase* (days 1 and 2), we fed bees the two unadulterated pollens and recorded the volume consumed of each of them. In the *training phase* (days 3 and 4), we adulterated *B. napus* pollen with linoleic acid, amygdalin or quinine. To control for the effect of botanical origin on learning, we replicated the experiment using *D. tenuifolia* as the adulterated pollen and *B. napus* as the unadulterated pollen of the *training phase*. Controls received both pollens unadulterated during this phase. Here, we also measured consumption of both pollens. During the *testing phase* (days 5 and 6), caged bees received unadulterated pollens. Again, we registered consumption. Based on the volume (ml) of pollen consumed from each syringe during the final 24 h of each phase, we calculated the Standardized Consumption (*SC*) of *B. napus* or *D. tenuifolia* pollen. For the *B. napus* series, *SC* was defined as the volume of *B. napus* pollen consumed relative to the total *B. napus* and *D. tenuifolia* pollen consumed. For the *D. tenuifolia* series, *SC* was defined as the volume of *D. tenuifolia* pollen consumed relative to the total pollen consumed. The *SCs* calculated for the *initial phase* allowed us to estimate the bees’ initial preferences for the two pollens and were later used in the statistical analysis to account for variations among bee cages (see “statistics” section). The *SCs* obtained for the *training phase* allowed us to confirm that the substances we used were effective for modifying pollen quality because caged bees that experienced deterrents avoided the adulterated pollen more than control groups and more than those that experienced linoleic acid (Table S1). Finally, the *SCs* obtained for the *testing phase* were compared among the four treatments (Linoleic acid, Amygdalin, Quinine, and Control) to answer whether positive or negative experiences with adulterated pollens lead to memories that bias consumption preferences.

### Experiment 2: Testing learned olfactory-based orientation preferences to odours associated with a deterrent-adulterated pollen

Here we determined whether odours associated with pollen could be learned to predict resource quality. We fed young caged bees with amygdalin-adulterated pollen scented with Linalool (LIO) or Nonanal (NONA) and measured their orientation response when exposed to the experienced odour and a novel one in a Y-maze^6,32^. LIO and NONA are neutral scents present in flowering plants^47^ and have been widely used as olfactory stimuli in learning and memory experiments. Because the time invested at the area of an odour may indicate the relevance of the cue for predicting rewards, we calculated the proportion of time spent in the arm scented with the odour experienced previously. Bees were collected in the same way as described for experiment 1; experimental phases were also similar, with only slight differences (Fig. 1B). During the *initial phase* (days 1 and 2), we fed caged bees multifloral pollen (from Paraná Delta Island). During the *training phase* (day 5), we fed them with one of the following options: (i) *B. napus* pollen unadulterated and unscented (Control), (ii) *B. napus* pollen adulterated with amygdalin and unscented (Amygdalin), (iii) *B. napus* pollen unadulterated and scented with 2 µl of LIO (Odour), or (iv) *B. napus* pollen adulterated with amygdalin (0.1 M) and scented with 2 µl of LIO (Odour + Amygdalin) (Fig. 1B). Whilst the Control treatment let us know the bees’ preferences for the tested odours, the Amygdalin treatment controlled for any effect of amygdalin on the bees’ odour response.

In this experiment, the procedure included a *pre-testing phase* (day 6; Figs. 1B; S1) in which we connected each cage to the Y-maze through a tube, allowing bees to explore the unscented device for 24 h and to become familiarized with it. During the *testing phase* (day 7) we scented one of the arms with LIO and the other arm with NONA. We released individual bees one at a time at the entrance channel of the Y-maze, which was not scented. Once a bee chose one of the arms (head and thorax beyond the arm entrance), we measured the accumulated time (s) spent in each arm from a total test time of 2 min. If a bee did not choose any arm within 1 min, it was excluded from the experiment. We tested each bee once. To reduce the effect of position on the bee’s orientation, we cleaned the Y-maze between trials and interchanged the sides of the odours.

We delivered the odours by means of a constant airflow (15 ml/s) that passed through a pair of 1 ml syringes containing a piece of filter paper with 4 µl of LIO or NONA (as novel odour) connected to the top of the arms. An extractor avoided odour contamination near the device. The proportion of time spent in the arm that presented the experienced odour was used as the response variable, calculated as the total time (s) spent in the experienced scent arm / total time (s) spent in both arms of the Y-maze. To control for the effect of odour identity on learning, we repeated the experiment using NONA as the experienced cue and LIO as the novel one.

### Experiment 3: Testing learned preferences in colonies that experienced changes in the quality of the offered pollens

As in experiment 1, but using colonies instead of cages, we tested the extent to which pollen consuming bees adjust their preferences within the social context of the hive. In this experiment, we used seven monofloral pollens (*Helianthus annus* L., *Salix* sp., *Rubus* sp., *Diplotaxis tenuifolia* (L.) DC., *Brassica napus* L., *Eucalyptus* sp., *Carduus* sp.) that were randomly assigned in pairs to each hive. We placed syringes with monofloral pollens between the central frames of the hive following the same temporal scheme used for experiment 1 (Fig. 1C). For the *initial phase*, we offered and measured the initial consumption of the two unadulterated monofloral pollens. For the *training phase*, we offered each colony with one of the two pollens adulterated with quinine, amygdalin, or linoleic acid (Fig. 1C). The control group of the colonies received both unadulterated pollens. In the colonies treated with linoleic acid, amygdalin, or quinine, the pollen adulterated within each pair was determined randomly, so that the effect of the treatment was independent of any specific pollen combination. For the *testing phase*, we offered and measured the consumption of both unadulterated pollens. As in experiment 1, we evaluated consumption preferences by means of the *SCs* of the pollen that had been previously adulterated. Because our intention was to determine the effect of the treatments was independent of the pollen used, and not to analyse differences in the identity of the pollen that conformed each pair, each combination was incorporated into the analysis as the consumption of the pollen that had been adulterated and the consumption of the pollen that had not been adulterated. *SCs* for the *initial phase* were used to account for variations among colonies in the analysis (see "statistics"). *SCs* for the *testing phase* were used to compare learned preferences among the four treatments (Linoleic acid, Amygdalin, Quinine, and Control).

### Statistics

All data were analysed using general linear models in R (http://www.R-project.org/). To assess differences in *SCs* of experiment 1, we explored the impact of treatment (Control, Linoleic acid, Amygdalin, and Quinine) and pollen identity (*B. napus* and *D. tenuifolia*) as fixed effects, and their interaction. We added initial preferences as covariate and total pollen consumption as offset. Differences in *SCs* registered in colonies (experiment 3) were analysed by means of a multiple linear regression with normal distribution. We analysed the effect of treatment (Control, Linoleic acid, Amygdalin, and Quinine) as fixed effect and we added total pollen consumption and the initial preferences as covariates. In all cases, homoscedasticity and normality assumptions were checked (Levene and Shapiro–Wilk tests, respectively). Tukey’s tests were used for contrasts using the multicomp package in R^48^. To test differences in the proportion of time spent in the arm that presented the experienced odour (experiment 2), we used a multiplicative generalized linear mixed model (GLMM) following a beta error distribution^49^. We considered treatment (four-level factor: Control, Amygdalin, Odour, and Odour + Amygdalin) and odour (two-level factor: LIO and NONA) as fixed effects, and each cage as a random factor. We used the ‘glmmTMB’ function of the ‘glmmTMB’ package^50^. Tukey’s multiple comparison tests were used when needed.

## Results

### Experiment 1: Effects of adulterated pollen on learned consumption preferences of nurse-aged bees

**Figure 2:**
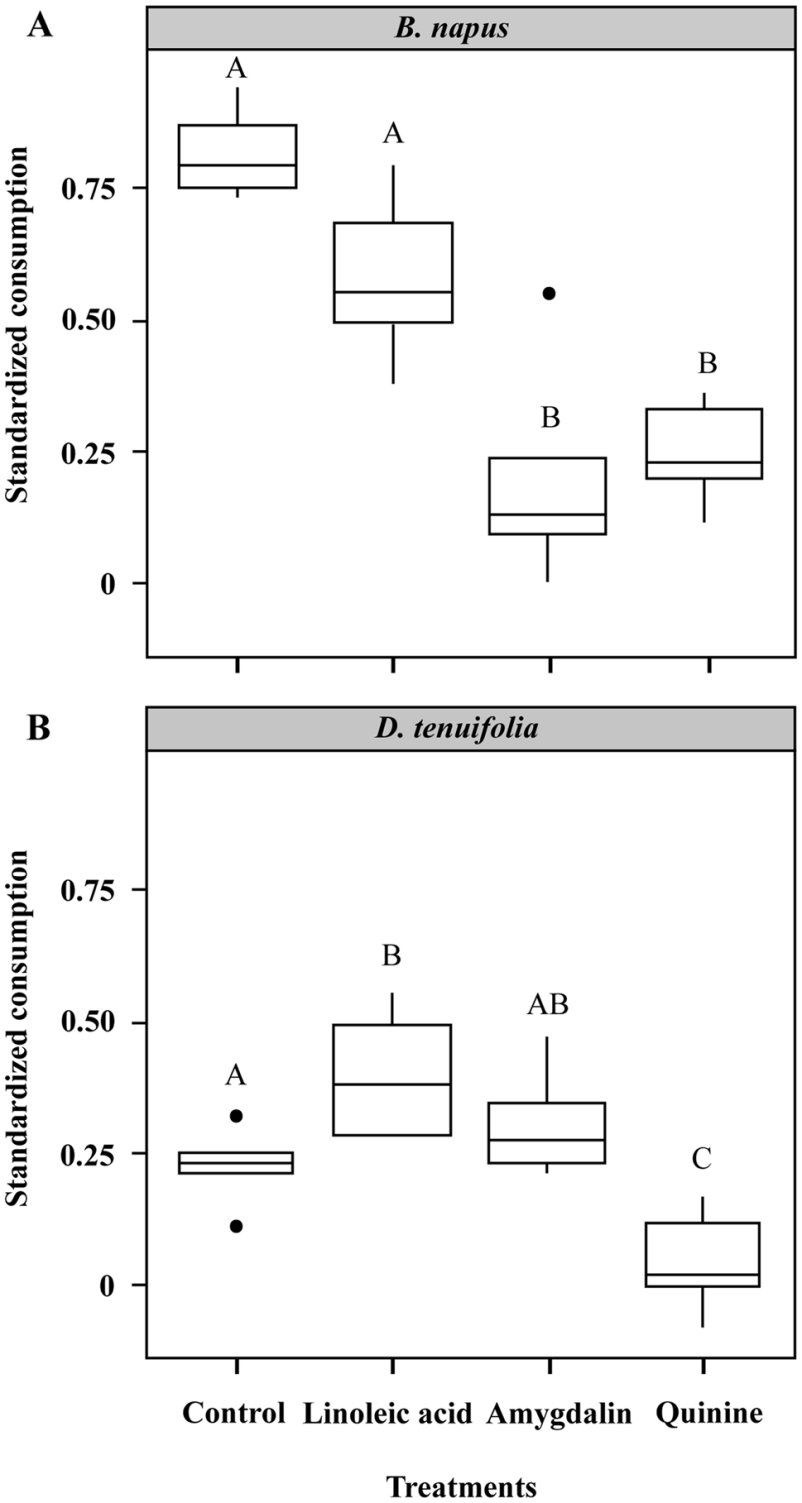
Effects of adulterated pollen on learned consumption preferences of nurse-aged bees. (**A**) Standardized consumption of *B. napus* was obtained after caged bees experienced *B. napus* pollen unadulterated (control) or adulterated with a phagostimulant (linoleic acid) or a deterrent substance (amygdalin or quinine). (**B**) Similarly, standardized consumption of *D. tenuifolia* was obtained after caged bees experienced *D. tenuifolia* pollen adulterated with linoleic acid, amygdalin, quinine, or unadulterated (control). The box plots show medians, quartiles, and 5th and 95th percentiles. Different letters indicate significant differences between treatments (*P* < 0.05).

Consumption preferences of the nurse-aged bees were affected by the identity and quality (adulterated or not) of pollen (F_3; 31_ = 9.8239; *P* = 0.0001; Fig. 2). The *SCs* of the *B. napus* series were lower after the offering of quinine-adulterated (0.244 ± 0.03, N cages = 5) and amygdalin-adulterated (0.201 ± 0.08, N cages = 4) pollen and were significantly different from the *SCs* of the Control (0.816 ± 0.06, N cages = 6) and Linoleic acid (0.581 ± 0.05, N cages = 5) treatments (Control—Amygdalin: t-ratio = 5.536; *P* < 0.0001; Control—Quinine: t-ratio = 4.171; *P* = 0.0012; Linoleic acid—Amygdalin: t-ratio = 4.678; *P* = 0.0003; Linoleic acid—Quinine: t-ratio = 3.677; *P* = 0.0047; Fig. 2A). *B. napus* pollen adulterated with linoleic acid did not affect the *SC* of nurse-aged bees (Control—Linoleic acid: t-ratio = 0.339; *P* = 0.9863; Fig. 2A) and no differences were detected between Amygdalin and Quinine (t-ratio = − 1.459; *P* = 0.4738).

*D. tenuifolia* pollen adulterated with quinine (0.04 ± 0.04, N cages = 5) decreased the *SCs*, compared to the control treatment (0.219 ± 0.03, N cages = 5), although not significantly (t-ratio = − 0.416; *P* = 0.9754; Fig. 2B). Interestingly, *D. tenuifolia* pollen adulterated with linoleic acid (0.393 ± 0.10, N cages = 6) significantly increased the *SCs* of nurse-aged bees (Linoleic acid—Control: t-ratio = − 3.175; *P* = 0.0169), whereas pollen adulterated with amygdalin (0.305 ± 0.12) did not affect the *SCs* (Amygdalin—Control: t-ratio = − 2.090; *P* = 0.1787; Fig. 2B). The nurse-aged bees preferred *B. napus* pollen rather than *D. tenuifolia* pollen, but the preference was similar when both pollens were adulterated with linoleic acid (Fig. 2). The impact of aversive substances such as quinine was high on *B. napus SCs*, but relatively low on *D. tenuifolia SCs*. In contrast, consumption preferences were strongly affected by supplementation of *D. tenuifolia* with linoleic acid but only slightly affected in the case of *B. napus*. Altogether, results show that young bees were able to change their consumption preferences according to the botanical origin and quality of pollen.

### Experiment 2: Olfactory cues associated with pollen influence orientation responses of nurse-aged bees

**Figure 3:**
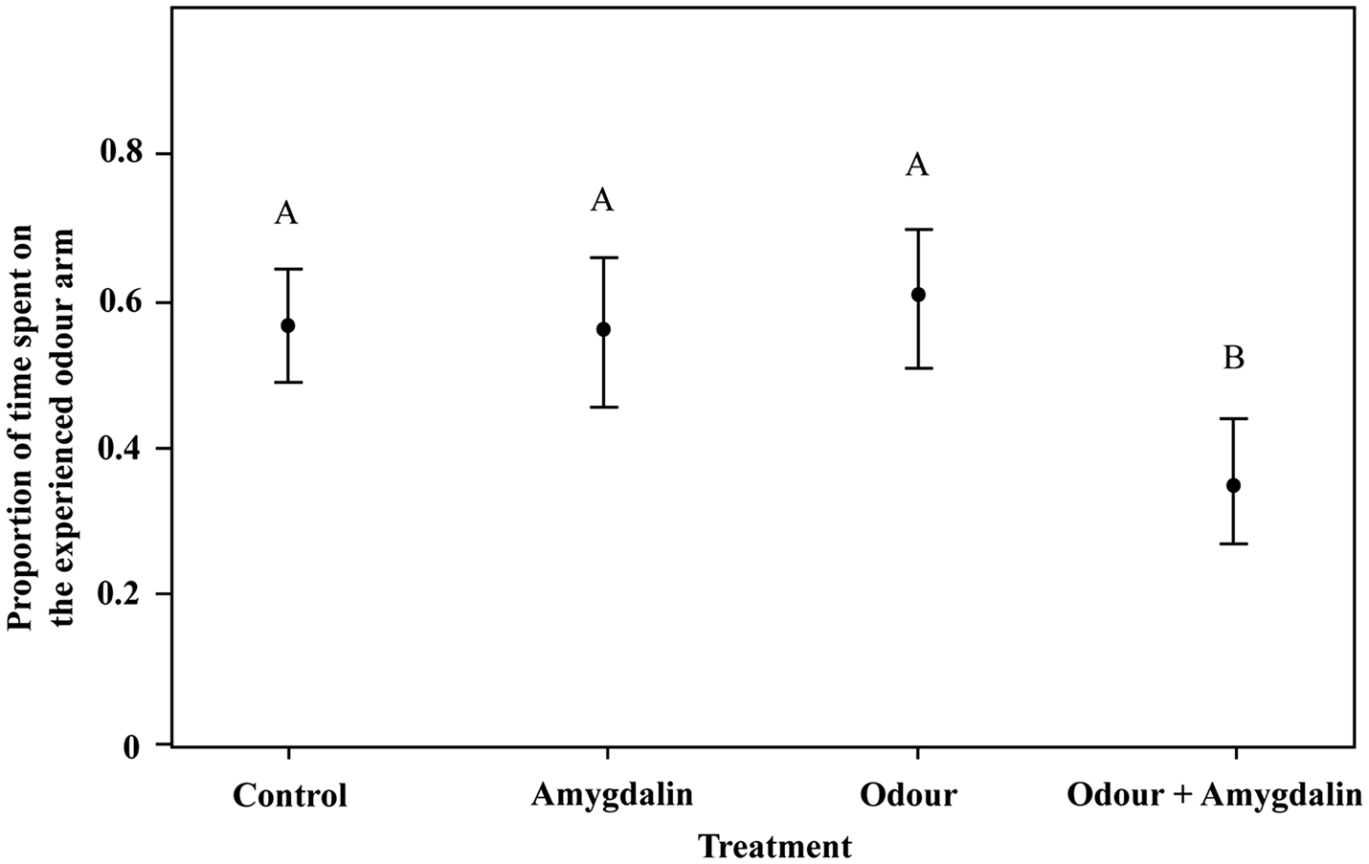
Effects of scented adulterated pollen on learned olfactory-based orientation response of nurse-aged bees. Proportion of time spent in the arm that presented the experienced odour as a function of four different treatments using pollen containing amygdalin as a deterrent and NONA or LIO as an additional olfactory cue. Because response was irrespective of odour identity, the data of LIO and NONA series were pooled. Black circles indicate the mean values and bars show the 95% confidence intervals. Different letters indicate significant differences between treatments (*P* < 0.05).

Our results indicate that olfactory cues associated with pollen influence orientation responses. We found that differences in the orientation response of nurse-aged bees were caused by the amygdalin-treated pollen (χ^2^ = 29.67, df = 3, *P* < 0.001, Fig. 3), but not by the odours (χ^2^ = 0.33, df = 1, *P* = 0.5655) nor by the interaction between treatment and odour (χ^2^ = 1.44, df = 3, *P* = 0.6947). The proportion of time that bees spent in the arm scented with the experienced odour was lower for caged bees treated with scented adulterated pollen (Odour + Amygdalin: 0.36 ± 0.0293, N cages = 14, N bees = 105, Fig. 3) than for bees that experienced the: (i) adulterated unscented (Amygdalin: 0.557 ± 0.0380, N cages = 10, N bees = 68, Fig. 3), (ii) unadulterated scented (Odour: 0.565 ± 0.0349, N cages = 12, N bees = 81) and (iii) unadulterated and unscented pollen (Control: 0.566 ± 0.0300, N cages = 12, N bees = 112, Fig. 3). The statistical analysis revealed that the adulterated scented treatment differed significantly from the rest of the treatments (Odour + Amygdalin—Amygdalin: t-ratio = 4.008; *P* = 0.004; Odour + Amygdalin—Control: t-ratio = 4.763; *P* < 0.0001; Odour + Amygdalin—Odour: t-ratio = 4.377; *P* = 0.0001, Fig. 3). Interestingly, the proportion of time spent in the arm that presented the experienced odour did not differ between adulterated and scented-treated cages, nor between the former and the control (Amygdalin—Odour: t-ratio = − 0.166; *P* = 0.9984; Control—Amygdalin: t-ratio = − 0.199; *P* = 0.9972; Fig. 3). Our results show that nurse-aged bees modify their orientation towards odours that they previously associated with distasteful and/or malaise experiences.

### Experiment 3: Effects of adulterated pollen on learned consumption preferences of nurse bees inside the hive

**Figure 4:**
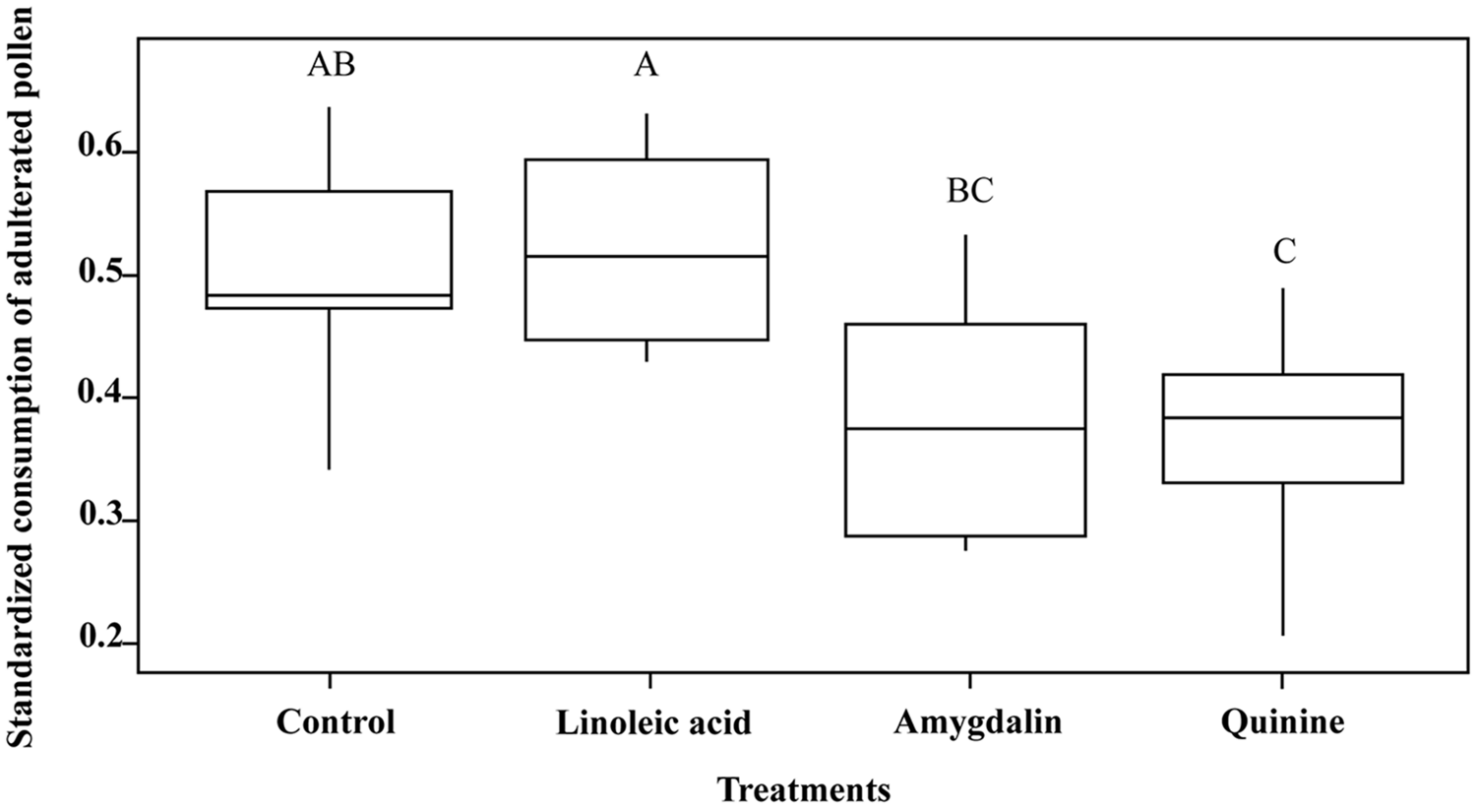
Effects of adulterated pollen on learned consumption preferences of nurse bees inside the hive. Standardized consumption of adulterated pollen calculated after colonies experienced one of the two pollens unadulterated (control) or adulterated by phagostimulant (linoleic acid) or deterrent (amygdalin or quinine) substances. The box plots show medians, quartiles, and 5th and 95th percentiles. Different letters indicate significant differences between treatments (*P* < 0.05).

Consumption preferences of colonies differed among treatments (Treatments: F_3; 17_ = 7.2482; *P* = 0.0024, Fig. 4). We found a significant reduction in the *SCs* (t-ratio = 3.614; *P* = 0.0104; Fig. 4) of colonies that experienced pollens adulterated with quinine (0.273 ± 0.0511, N colonies = 7), but not (t-ratio = 2.369; *P* = 0.1215) in the *SCs* of colonies that experienced pollens adulterated with amygdalin (0.389 ± 0.0435, N colonies = 6). On the other hand, those colonies that were fed pollen supplemented with linoleic acid showed an increase in the preference for the adulterated pollen (0.589 ± 0.0495, N colonies = 5), although it was not significant (t-ratio = − 0.851; *P* = 0.8293). Interestingly, the *SCs* of linoleic acid-treated colonies did differ from the *SCs* of amygdalin- and quinine-treated colonies (t-ratio = 3.049; t-ratio = 4.138; *P* = 0.0332, *P* = 0.0035, respectively, Fig. 4). Learned consumption preferences established in colonies fed with adulterated pollen were consistent with results observed in bee cages that experienced both adulterated and unadulterated pollens simultaneously (experiment 1).

## Discussion

Up to now, very little was known about the ability of nurse bees to learn and retain information about pollen. In this study, we provide evidence that their pollen-mediated responses can adaptively change based on learned information. Nurse bees that consume large quantities of pollen to feed the brood and colony mates, could benefit from learning and retaining the identity of the resources they experience as food. Learning of neutral stimuli associated with the pollen source (e.g., odours) might expand the type and amount of information to which young bees can respond. Inside the hive, nurse bees might use learned information to modify their pollen-related responses, making a further selection of the resources that have been incorporated into the nest.

### Generalization and discrimination of pollen-based stimuli

The interest in studying the ability of young bees to learn and retain cues from pollen is quite recent. A previous study^32^ showed that young bees fed with single monofloral pollen diets (Solanaceae species, *Taxodium* sp., or *Hypochaeris* sp.) did not bias the orientation response towards the specific pollen-based odour (i.e., bees chose the experienced and the novel monofloral pollen odours equally). In our study, we also did not detect differences in consumption preferences between *B. napus* and *D. tenuifolia* after nurse-aged bees had experienced the deterrent-adulterated *B. napus* alone during training (without the simultaneous offering of an alternative unadulterated monofloral pollen like in experiment 1; see Fig. S2). Furthermore, we observed that bees trained with amygdalin-adulterated *B. napus* pollen did not avoid the natural odour of *B. napus* pollen when confronted with a novel pollen odour in the Y-maze (Fig. S3). Lack of differences in those experiments may be due to generalisation, which may account for the extension of the predictive value of learned information about a source to a similar but different novel one^51–53^. Several studies using different experimental approaches^54,55^ have already found that honeybees can generalize pollen odours between taxonomically unrelated plants.

On the contrary, it has been shown that bees distinguish between pollen odours accurately if trained in a differential-like manner, for instance when one of the odours is presented rewarded and the other unrewarded^54^. Consistently, results from experiment 1 indicate that, if bees were to experience the adulterated and the unadulterated pollen simultaneously, they would be able to perceive and learn pollen-based cues of each pollen to achieve discrimination. Similarly, in the open colonies (experiment 3), nurse bees may have learned the single adulterated pollen offered during the *training phase* by discriminating it against the many other (but undetermined) pollens that entered the nest. Thus, our results indicate that, although bees tend to generalize pollen-based cues, they can discriminate among different pollens if learned together.

### Gustatory perception versus post-ingestion state induced by aversive and phagostimulant compounds

Among the substances we used to modify the quality of pollens, the deterrents quinine and amygdalin were the most effective in inducing memory formation when added to the highly preferred *B. napus* pollen. Quinine was also effective when added to *D. tenuifolia*. The strength of these memories positively correlates with the volume of adulterated pollens consumed during the *training phase* of our experiments (Tables S1–S3), for which we observed that consumption of amygdalin- and quinine-adulterated pollen decreased drastically compared with Control groups. Furthermore, *SCs* obtained for the *training phase* (Table S1) confirmed that bees did assess the aversive substance in pollen as they showed a clear avoidance for both pollens. Unfortunately, our experiments do not allow us to define the mechanism the bees use for the evaluation. Previous experiments testing quinine and amygdalin solutions on harnessed foraging bees demonstrated no pre- but a post-ingestive effect^44,56^ as bees associated gustatory cues with the aversive compounds after extended delays and not immediately (but also see^56^). By learning through the state induced after ingestion of deterrent substances, nurse bees could avoid inappropriate resources that foragers incorporate into the colony due to their lack of access to constituents that can only be assessed after pollen ingestion, thus enabling an additional step of pollen quality control after foraging.

### Behavioural responses of nurse bees to natural compounds and agrochemicals in pollen

Deterrent substances in pollen exert repulsive responses or induce distasteful and/or malaise experiences. Among them, secondary compounds such as nicotine in *Nicotiana tabacum* L. (tobacco) or amygdalin in *Prunus dulcis* (Mill.) D.A.Webb (almond)^17,18^ occur naturally in pollen. In addition, other artificial substances applied to fields might contaminate pollen. In particular, agrochemicals like herbicides or insecticides^58,59^ might be present in the pollen that foragers take back to the hive, affecting the health and functioning of the colony. Imidacloprid, for example, one of the most widely used insecticides globally, impacts the behaviour of young honeybees by affecting information acquisition during associative learning and leading to a decrease in sucrose sensitivity^60,61^. On the other hand, glyphosate, a broad-spectrum herbicide used for weed control, did not interfere with simple learning, but did reduce olfactory discrimination in young workers. Interestingly, bees fed sugar solution with glyphosate consumed less than the control group, suggesting that glyphosate is somehow detected pre- or post-ingestion^61^. Given the ability observed in young bees to avoid pollen experienced with noxious compounds, we wonder whether bees could also avoid pollen contaminated with pesticides to mitigate the deleterious effects of these compounds in colonies that pollinate in agro-ecosystems.

### Could nurse bees influence foraging decisions?

Pollen preferences might not be the same in foragers and nurse bees. While some pollens belonging to *Kallstroemia grandiflora* Torr. ex A. Gray and *Baccharis sarothroides* A. Gray were reported to be collected frequently by foragers but accepted seldomly by the young in-hive bees, other pollens present in *Ambrosia* sp. and *Taraxacum officinale* Weber ex F.H.Wigg, were shown to be readily collected by foragers and consumed by young bees despite providing poor nutrition^62^. Without a definite explanation in this regard, we expect that both foragers and nurse workers could be involved in the process of pollen selection, based on their distinct access and type of manipulation of the resource during their tasks.

Considering that foraging is influenced by interactions that occur within the nest^7,63^, an important question is whether the selections young bees make impact pollen foragers’ decisions or not. Evidence leads us to speculate that even if foragers themselves are incapable of making a complete assessment of pollen, there are mechanisms operating at the colony level that modify their foraging preferences. In this regard, foragers tend to actively avoid some low-quality pollens (e.g., cucumber, cotton, or cattails) that are highly available in the environment^16,64,65^. Moreover, selection of pollens containing essential amino acids and essential fatty acids that were deficient in the colony has also been observed^66,67^. Thus, we reason that foragers’ decisions may be influenced by in-hive bees that, as demonstrated here, could participate in pollen selection among pollens available in the nest (e.g., by learning to avoid pollens less suitable for colonies). The extent to which the responses of young bees and the modifications that they produce in the nest while choosing pollen influence preferences of foragers remains to be investigated.

### Final remarks

Loss of floral diversity due to changes in land use and modern agricultural practices causes a reduction in pollen availability that negatively affects the nutritional status of colonies^68–73^. Pollens with agrochemicals exert deleterious effects on bees and compromise colony development. In this scenario, increasing the knowledge about mechanisms that enable honeybees to respond to environmental fluctuations in pollens and to sudden changes in their quality, is a priority. Here, we studied the behavioural responses of bees that might participate in the selection of pollen inside the hive. Taking into account bees’ cognitive abilities together with aspects of their social organisation, our study indicates that, based on learning, nurse bees can adaptively change their pollen preferences to the most suitable/required pollens.

## Supplementary Information

Supplementary Information 1.

Supplementary Information 2.
