# Supplementary material for: Young honeybees show learned preferences after experiencing adulterated pollen
Source: Sci Rep. 2021 Dec 2;11:23327. doi: 10.1038/s41598-021-02700-6 (PMC8640054; doi:10.1038/s41598-021-02700-6)
Supplement: Supplementary file 2 — Supplementary Information 2. [file 41598_2021_2700_MOESM2_ESM.pdf]

Supporting information

Figure S1

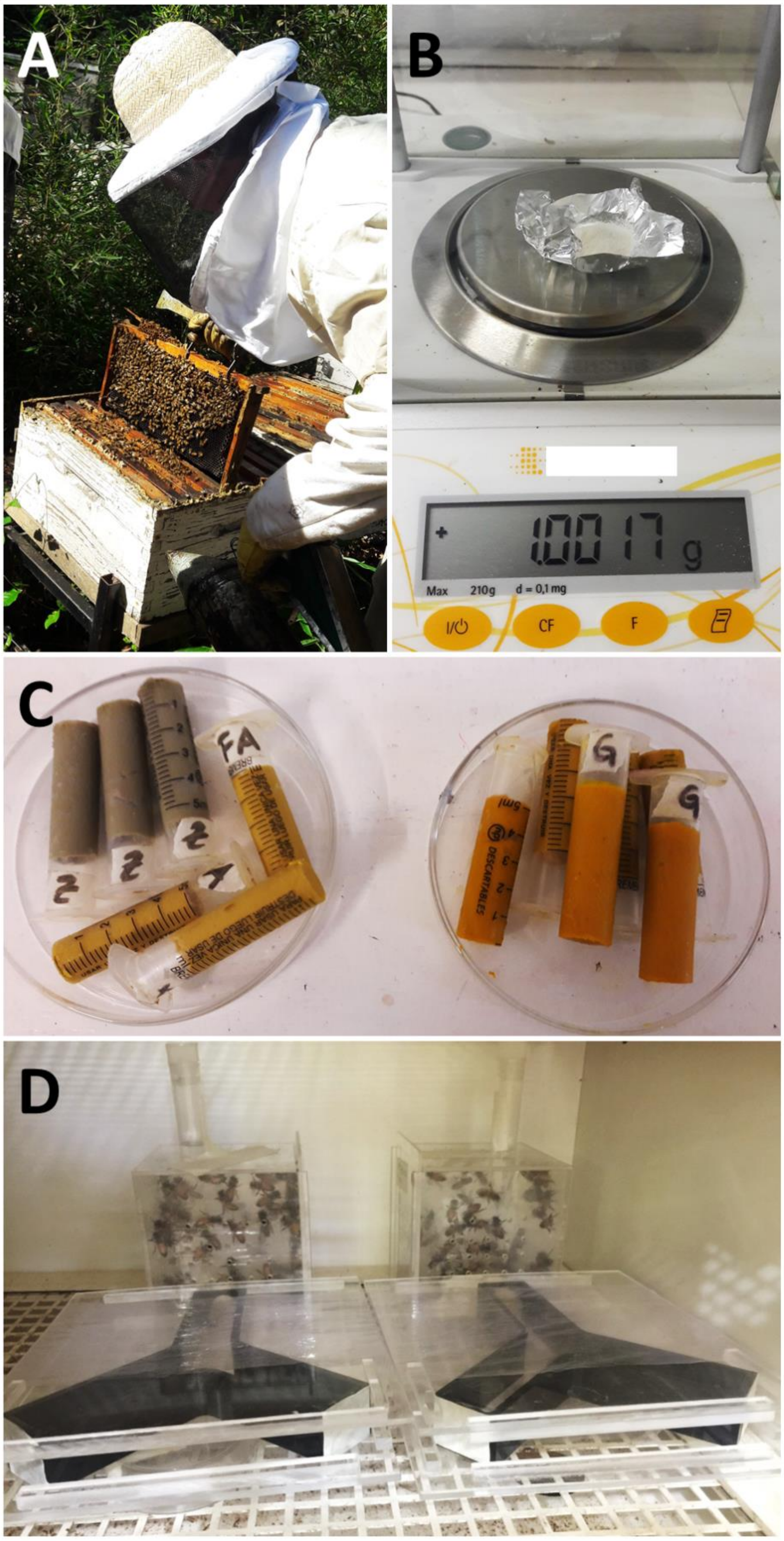

Figure S1. Photos of the experimental designs. **A**) Brood frames obtained from a conventional hive in the Experimental Field of the School of Exact and Natural Sciences of the University of Buenos Aires. **B**) Weighing of a deterrent substance that will be added to monofloral pollen in order to modify its quality. **C**) Different monofloral pollens in bee bread-like paste offered in tipless 5 ml graduated syringes. **D**) *Pre-testing phase* in experiment 2.

Experiment S1: Effects of a single adulterated pollen on learned consumption preferences of nurse-aged bees.

Here we studied young bees' ability to learn and retain pollen information using a single adulterated pollen during training (i.e., the learning procedure involved *B. napus* pollen; Fig. S2A). For the first four days of adult life, we fed the bee cages multifloral pollen *ad libitum* from Paraná Delta Island (*initial phase*). On the fifth day and for 24 hours (*training phase*), we replaced multifloral pollen by 2 ml of *Brassica napus* pollen that had been adulterated with amygdalin or LiCl (Fig. S2A). Control cages received unadulterated *B. napus* pollen. For the next 48 hours (*testing phase*), bee-cages received the experienced *B. napus* pollen but also the novel *Diplotaxis tenuifolia* pollen, both unadulterated and presented in one 5 ml syringe each. We registered the volume (ml) of pollen consumed from each syringe during the last 24 h of the *testing phase* and calculated the Standardized Consumption (*SC*) of *B. napus* pollen, defined as the consumption of *B. napus* relative to the total pollen consumption of *B. napus* plus *D. tenuifolia* pollen. To test differences in the *SCs* of cages, we carried out the same analysis as experiments 1 and 3. Then, we explored the impact of treatment (Control, LiCl and Amygdalin) as a fixed effect and added total pollen consumption as offset.

Consumption preferences were similar in cages subjected to different treatments (Fig S1B). Consequently, we did not detect any significant differences among *SCs* obtained in Amygdalin, LiCl and Control cages (Treatments*:* $F_{2;\,20} = 0.5267$; $P = 0.5985$). The *SCs* in both

Control and Amygdalin groups were very similar and revealed a slight preference of *B. napus* pollen over *D. tenuifolia* pollen (Control: 0.66 ± 0.11, N cages = 9; Amygdalin: 0.72 ± 0.13, N cages = 7; Fig. S1B). Caged-bees that experienced *B. napus* pollen adulterated with LiCl exhibited the lowest *SCs* (LiCl: 0.52 ± 0.16, N = 7), yet such values did not differ from the Control or the Amygdalin group.

A

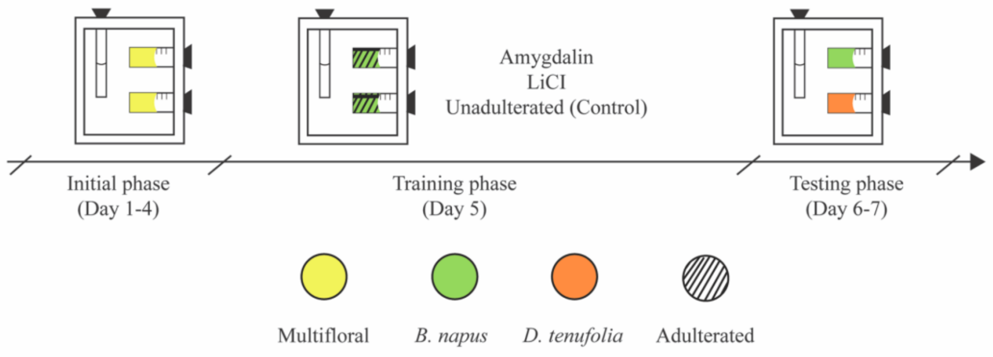

B

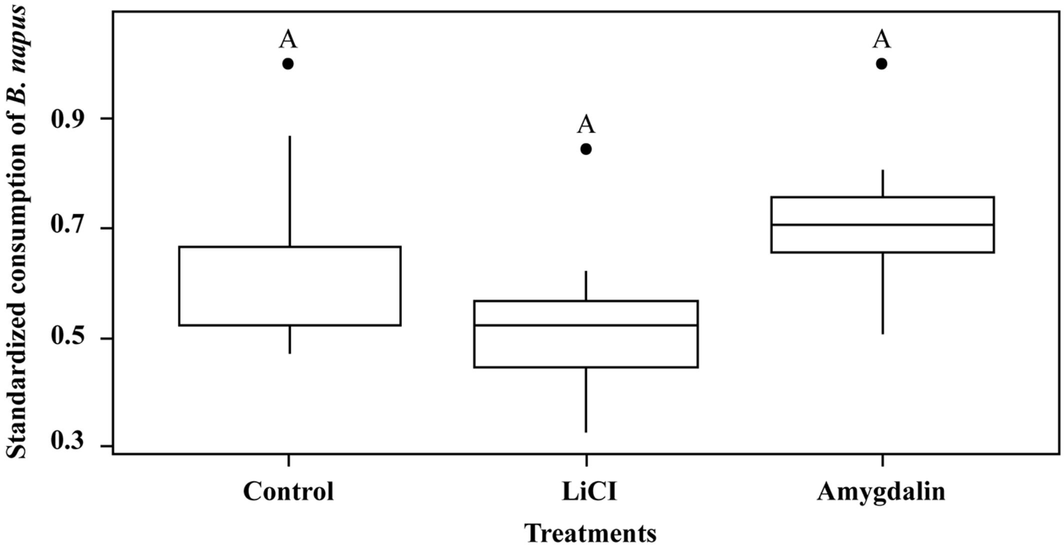

Fig S2. Effects of adulterated pollen on consumption preferences of nurse-aged bees that experienced changes in the quality of a single offered pollen. A) Schematic schedule of experiment S1 over the whole experimental period. **B)** Standardized Consumption of *B. napus* pollen after cages experienced one of the two pollens unadulterated (Control) or adulterated by a deterrent substance (LiCl or amygdalin). The box plots show medians, quartiles and 5th and 95th percentiles. The same letter indicates no significant differences between treatments.

Experiment S2: Orientation response to the volatiles of pollen experienced adulterated by amygdalin.

Here, we tested whether natural odours emitted by pollen (henceforth: pollen-based cues) could be learned by young bees and whether this biases the orientation response in a Y-maze. Bee-cages were fed bee-collected *B. napus* pollen that had been previously adulterated with amygdalin (0.1 M, Fig. S3A). Pollen volatiles of *B. napus* pollen (experienced) and *D. tenuifolia* pollen (novel) were swept up from small pollen samples (10 corbicules) contained inside 10 ml

syringes. Control treatments with unadulterated pollen were included. We measured the proportion of time spent on each arm of the maze during the test and we carried out the same analysis as in experiment 2. Then, we considered treatment (two-level factors corresponding to Control and Amygdalin) as fixed effects, and each cage as a random factor.

Mean proportion of time spent on the arm that presented the experienced natural odours emitted by *B. napus* pollen for adulterated treatment (Amygdalin: 0.703 ± 0.0442, N cages = 4, N bees = 40) was similar to the control treatment (Control: 0.724 ± 0.0430, N cages = 4, N bees = 39; Fig. S3B). When the natural bee-collected pollen volatiles were used as olfactory cues in the Y-maze, the proportion of time spent on the experience odour arm did not differ between the adulterated and the control group (Amygdalin - Control: χ^2=0.136, df=1 P = 0.712, Fig. S3B).

A

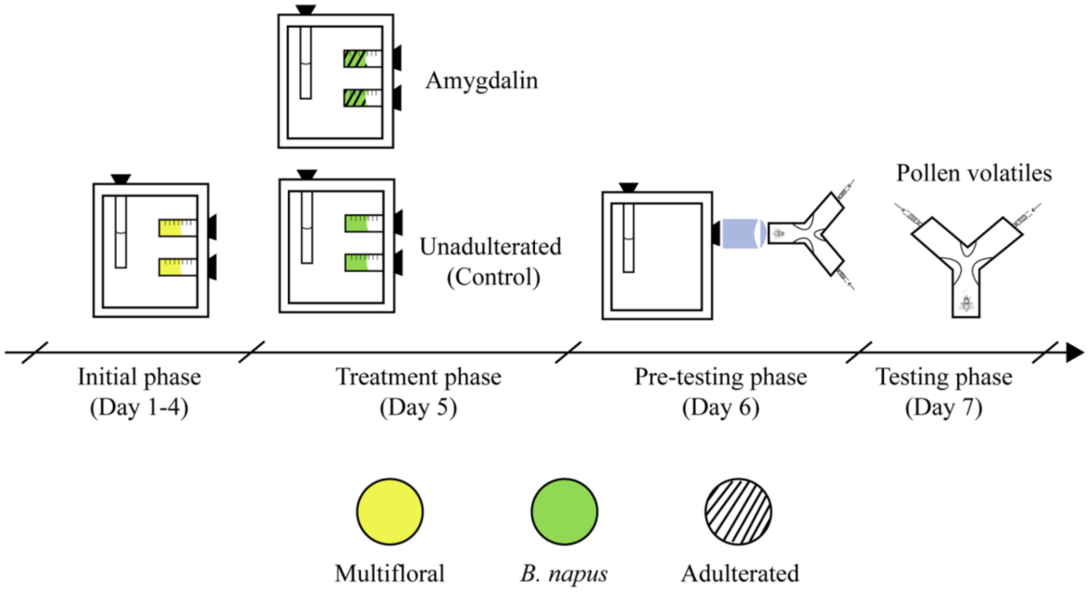

B

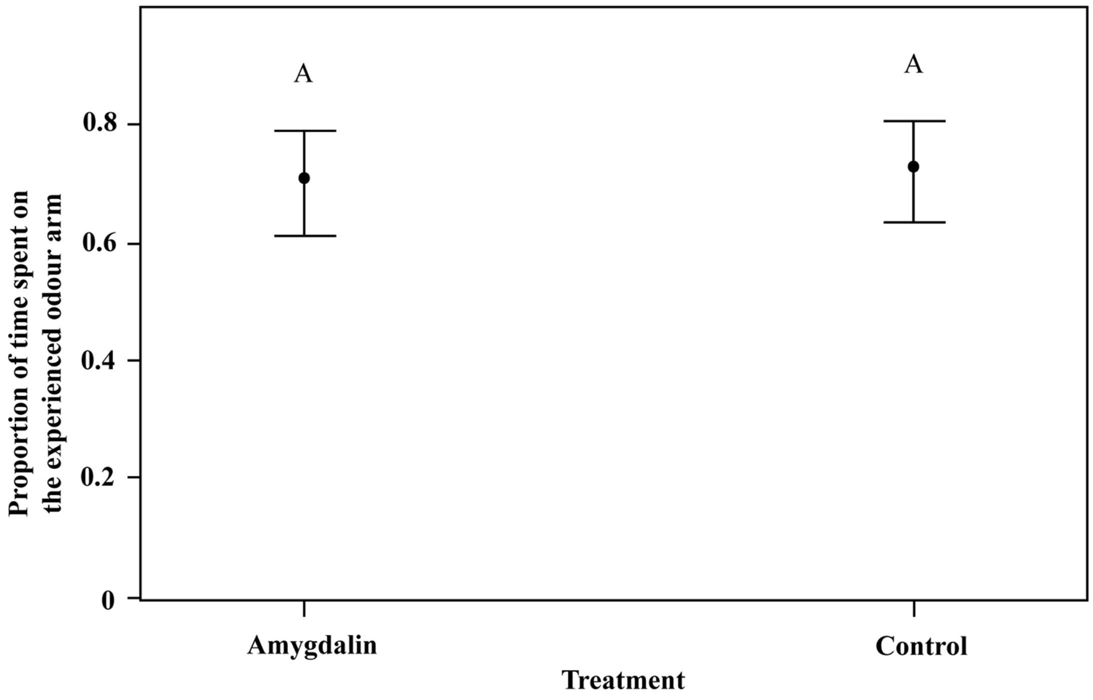

Fig S3. **Orientation response to the volatiles of pollen experienced adulterated by amygdalin**. **A)** Schematic schedule of experiment S2 over the whole experimental period. **B)** Proportion of time spent on the arm that presented the odour of the experienced pollen (i.e., volatiles swept up from small pollen samples). Black circles indicate the mean values and bars show the 95% confidence intervals. Similar letter indicates absence of statistical differences between treatments.

Table S1. Consumption and preferences for pollens in caged bees that experienced changes in the quality of the offered pollens. Volume (ml) consumed of *B. napus* pollen and *D. tenuifolia* pollen before (*initial phase*), during (*training phase*) and after (*testing phase*) *B. napus* (**A**) or *D. tenuifolia* (**B**) pollen were offered adulterated (Linoleic acid, Amygdalin or Quinine) or unadulterated (Control).

A.

B. napus pollen series	*n*	*Initial phase*			*Training phase*			*Testing phase*		
		**Consumption (ml)**		***SC***	**Consumption (ml)**		***SC***	**Consumption (ml)**		***SC***
		D. tenuifolia	*B. napus*		*D. tenuifolia*	*B. napus*		*D. tenuifolia*	*B. napus*	
Control	*6*	0.13±0.03	0.30±0.06	0.66±0.11	0.08±0.03	0.50±0.04	0.88±0.05	0.08±0.02	0.40±0.05	0.82±0.06
Linoleic acid	*5*	0.33±0.05	0.18±0.10	0.30±0.14	0.27±0.05	0.24±0.09	0.44±0.11	0.12±0.03	0.16±0.03	0.58±0.05
Amygdalin	*4*	0.19±0.09	0.41±0.06	0.71±0.12	0.46±0.05	0.01±0.01	0.02±0.02	0.33±0.06	0.11±0.07	0.20±0.08
Quinine	*5*	0.37±0.08	0.31±0.07	0.46±0.08	0.35±0.06	0.04±0.02	0.10±0.04	0.25±0.04	0.08±0.01	0.24±0.03

B.

D. tenuifolia pollen series	*n*	*Initial phase*			*Training phase*			*Testing phase*		
		**Consumption (ml)**		***SC***	**Consumption (ml)**		***SC***	**Consumption (ml)**		***SC***
		D. tenuifolia	*B. napus*		*D. tenuifolia*	*B. napus*		*D. tenuifolia*	*B. napus*	
Control	*5*	0.17±0.06	0.37±0.04	0.30±0.09	0.12±0.03	0.62±0.07	0.16±0.03	0.11±0.02	0.38±0.04	0.22±0.03
Linoleic acid	*6*	0.22±0.05	0.13±0.07	0.74±0.13	0.18±0.03	0.41±0.06	0.31±0.05	0.13±0.04	0.18±0.04	0.39±0.10
Amygdalin	*4*	0.28±0.09	0.25±0.10	0.54±0.21	0.06±0.02	0.40±0.09	0.16±0.07	0.13±0.06	0.23±0.01	0.30±0.12
Quinine	*5*	0.21±0.10	0.31±0.08	0.33±0.13	0.04±0.02	0.58±0.09	0.07±0.03	0.01±0.01	0.29±0.05	0.04±0.04

The tables show the mean pollen consumption obtained in several bee-cages consisting of 80 known-age individuals each. Numbers in brackets indicate the sample size in each treatment.

Table S2. Consumption and preferences for pollens in colonies. Volume (ml) consumed of two different monofloral pollens (*Pollen A* and *B*), before (*initial phase*) and after (*testing phase*) *Pollen A* was offered adulterated (Linoleic acid, Amygdalin or Quinine) or unadulterated (Control) during the *training phase*. Consumption of *Pollen A* was also quantified during training.

**Treatment**	***n***	***Initial phase***			***Training phase***	***Testing phase***		
		**Consumption (ml)**		***SC***	**Consumption (ml)**	**Consumption (ml)**		***SC***
		Pollen A	*Pollen B*		*Pollen A*	*Pollen A*	*Pollen B*	
Control	*6*	3.53±0.47	3.50±0.45	0.50±0.04	6.53±0.70	3.03±0.42	2.97±0.28	0.50±0.03
Linoleic acid	*5*	1.98±0.58	2.00±0.56	0.50±0.03	6.42±0.62	2.74±0.71	2.44±0.48	0.52±0.05
Amygdalin	*6*	3.27±0.75	2.83±0.54	0.52±0.03	5.27±0.98	2.62±0.76	3.70±0.41	0.38±0.09
Quinine	*7*	4.40±0.39	2.89±0.23	0.6±0.02	7.08±0.68	2.40±0.55	3.51±0.43	0.38±0.05

The table shows the mean pollen consumption obtained in several ten-frame langstroth hives that remained open during the experiment. Numbers in brackets indicate the sample size in each treatment.

Table S3. Consumption of pollen in caged bees that experienced changes in the quality of a single offered pollen. Volume (ml) of pollen consumed during the *training phase*, while caged-bees were fed the *B. napus* pollen either adulterated by a deterrent substance (LiCl or Amygdalin) or unadulterated (Control), and during the *testing phase*, while caged-bees were fed *B. napus* pollen and *D. tenuifolia* pollen, both unadulterated.

**Treatment**	***n***	**Consumption (ml)**		
		Training phase	***Testing phase***	
		B. napus	*D. tenuifolia*	*B. napus*
Control	8	0.39±0.07	0.11±0.04	0.21±0.05
LiCl	7	0.30±0.09	0.14±0.04	0.17±0.05
Amygdalin	7	0.13±0.03	0.06±0.02	0.25±0.06

The table shows the mean pollen consumption obtained in several bee-cages consisting of 80 known-age individuals each.
